# Effects of Astragaloside IV and Formononetin on Oxidative Stress and Mitochondrial Biogenesis in Hepatocytes

**DOI:** 10.3390/ijms26020774

**Published:** 2025-01-17

**Authors:** Quoc-Anh Tran, Grant Van Tran, Sanel Velic, Hou-Mai Xiong, Jaspreet Kaur, Zuhurr Moosavi, Phuong Nguyen, Nhi Duong, Vy Tran Luu, Gurjot Singh, Tram Bui, Melanie Rose, Linh Ho

**Affiliations:** College of Pharmacy, California Northstate University, Elk Grove, CA 95757, USA

**Keywords:** astragaloside, formononetin, SIRT3 modulators, oxidative stress, oxidative injury, *tert*-butyl hydrogen peroxide (*t*-BHP), mitochondrial sirtuins, SIRT3

## Abstract

Over-accumulation of reactive oxygen species (ROS) causes hepatocyte dysfunction and apoptosis that might lead to the progression of liver damage. Sirtuin-3 (SIRT3), the main NAD+-dependent deacetylase located in mitochondria, has a critical role in regulation of mitochondrial function and ROS production as well as in the mitochondrial antioxidant mechanism. This study explores the roles of astragaloside IV (AST-IV) and formononetin (FMR) in connection with SIRT3 for potential antioxidative effects. It was shown that the condition of combined pre- and post-treatment with AST-IV or FMR at all concentrations statistically increased and rescued cell proliferation. ROS levels were not affected by pre-or post-treatment individually with AST-IV or pre-treatment with FMR; however, post-treatment with FMR resulted in significant increases in ROS in all groups. Significant decreases in ROS levels were seen when pre- and post-treatment with AST-IV were combined at 5 and 10 μM, or FMR at 5 and 20 μM. In the condition of combined pre- and post-treatment with 10 μM AST-IV, there was a significant increase in SOD activity, and the transcriptional levels of Sod2, Cat, and GPX1 in all treatment groups, which is indicative of reactive oxygen species detoxification. Furthermore, AST-IV and FMR activated PGC-1α and AMPK as well as SIRT3 expression in AML12 hepatocytes exposed to *t*-BHP-induced oxidative stress, especially at high concentrations of FMR. This study presents a novel mechanism whereby AST-IV and FMR yield an antioxidant effect through induction of SIRT3 protein expression and activation of an antioxidant mechanism as well as mitochondrial biogenesis and mitochondrial content and potential. The findings suggest these agents can be used as SIRT3 modulators in treating oxidative-injury hepatocytes.

## 1. Innovation

**Figure 1:**
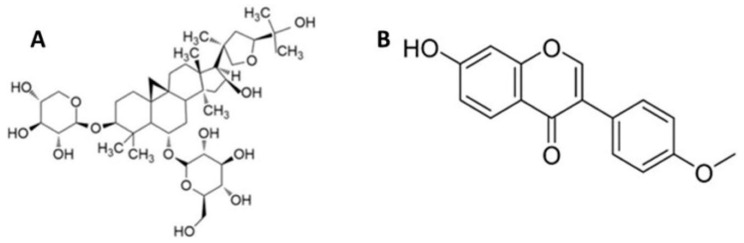
Chemical structure of (**A**) astragaloside IV (a cycloartane-type triterpene glycoside); and (**B**) formononetin (O-methylated isoflavone).

Over-accumulation of reactive oxygen species (ROS) can lead to hepatocyte dysfunction and might progress to liver damage. Sirtuin-3 is the major deacetylase enzyme in mitochondria that has an important role in modulating mitochondrial antioxidant mechanism and ROS production. Astragaloside (AST-IV, Figure 1A) and formononetin (FMR, Figure 1B) were found to activate Sirtuin-1 for improving fibrosis of mouse podocytes and reducing inflammation and mucus formation in airway epithelial cells derived from a patient with cystic fibrosis. This study revealed that AST-IV and FMR activate mitochondrial Sirtuin-3 and an antioxidant mechanism, as well as mitochondrial biogenesis and a function for their antioxidative effect, protecting hepatocytes from oxidative injury induced by *t*-BHP.

## 2. Introduction

Oxidative stress and mitochondrial dysfunction have been found to contribute to the initiation and progression of clinical and experimental liver damage [1,2]. Over-accumulation of reactive oxygen species (ROS) causes hepatocyte dysfunction and apoptosis, infiltration of monocytes into the liver, and activation of hepatic stellate liver cells, resident macrophages (Kupffer cells), and liver sinusoidal endothelial cells in the progression of liver damage [3,4]. Elimination of excessive ROS is one of the effective strategies to protect liver cells. Mitochondrial dysfunction impairs redox homeostasis and metabolic adaptation, leading to abnormalities in the structure and function of the liver [5,6,7]. SIRT3, the main NAD+-dependent deacetylase located in mitochondria, has a critical role in regulation of mitochondrial function and ROS production as well as the mitochondrial antioxidant mechanism [8,9]. SIRT3 deficiency increases superoxide and reactive oxygen species (ROS) associated with low activity of the antioxidant enzyme MnSOD in live tissue [10,11]. In addition to MnSOD, SIRT3 also targets and activates mitochondrial superoxide dismutase 2 (SOD2) [11] and isocitrate dehydrogenase 2 (IDH2) via deacetylation [8,9] to enhance antioxidative capacity. Isocitrate dehydrogenase (IDH) catalyzes the oxidative decarboxylation of isocitrate into alpha-ketoglutarate and produces NADPH and increases the ratio of reduced GSH to the oxidized form GSH (GSSG), antagonizing ROS-mediated cell injury [12,13]. As oxidative stress increases, the oxidized form (GSSG) becomes the predominant molecule [12]. Moreover, glutamate dehydrogenase (GDH), an enzyme that catalyzes the conversion of glutamate to α-ketoglutarate, is deacetylated and activated by SIRT3, promoting the production of NADPH, protecting cells against oxidative stress [13]. SIRT3 promotes mitochondrial biogenesis and maintains mitochondrial structural integrity by upregulating peroxisome proliferator-activated receptor γ coactivator 1α (PGC-1α) [14,15]. Taken together, from all these known data, mitochondrial SIRT3 has a key role in the antioxidative defense mechanism via its deacetylation and its potential activators are beneficial for treatment of oxidative stress-induced liver damage.

In addition, SIRT3 has a role in liver fibrosis, thus is a potential therapeutic approach for its treatment. SIRT3 protects hepatocytes from oxidative stress by scavenging reactive oxygen species (ROS) and maintaining mitochondrial integrity [16]. Similarly, SIRT3 reduces the inflammatory infiltration of macrophages and decreases the expression of inflammatory factors by inhibiting the NOD-like receptor thermal protein domain-associated protein 3 (NLRP3) inflammasome [17]. Furthermore, SIRT3 inhibits the activation of hepatic stellate cells (HSCs) by mediating the downstream signaling pathway of transforming growth factor-β (TGF-β)-Smad in liver fibrosis [18]. SIRT3 plays a key role in the development of liver disease and is expected to be a potential target for the prevention and treatment of liver fibrosis.

AST-IV is a saponin molecule derived from *Astragalus membranaceus*, a herbal plant in the family Fabaceae [19]. It exhibits a wide range of pharmacological properties including antioxidative stress, hepatoprotection, and anti-inflammation in the treatment of many diseases such as liver fibrosis and non-alcoholic fatty liver disease [20,21,22,23]. AST-IV was found to have antioxidant activity by ameliorating the mitochondrial disorder and restoring the expression of Nrf2 and TFAM in oxidative stress-induced diabetic kidney injury [24]. In addition, it improves ethanol-induced liver injury by inhibiting oxidative stress via suppression of NF-κB signaling [25] and inhibition of the NLRP3/caspase-1 pathway [26].

Formononetin (FMR) is a secondary metabolite of flavonoids present in legumes and graminaceous plants such as *Astragalus mongholicus* Bunge [Fabaceae; Astragali radix] and *Avena sativa* L. [*Poaceae*] [27]. FMR may decrease oxidative stress in induced liver injury through promoting the PHB2/PINK1/Parkin-mediated mitophagy pathway [28] and increased antioxidant activity in H_2_O_2_-treated Chang Liver cells [29]. In addition, FMR was reported to reduce ritonavir-induced hepatotoxicity via inhibiting ROS formation, thus mitigating its side effects and enhancing its safety [30]. Interestingly, FMR was proved to remarkably attenuate oxidative stress by decreasing the ROS level in the airways of allergic asthma-induced mice [31].

Furthermore, AST-IV and FMR were found to enhance expression of Sirtuin-1 (SIRT1) [32,33,34,35]. AST-IV increased SIRT1 expression when used on mouse podocytes, which reversed renal fibrosis and improved renal function in the experimental mice compared to mice that were not treated with AST-IV [32]. Another study, from 2018, treated mice with AST-IV while analyzing autophagy of mesangial cell activation in diabetic kidney disease [33]. Under AST-IV treatment, SIRT1 expression increased, enhancing autophagy and improving renal function and fibrosis [33]. Treatment with an SIRT1 inhibitor showed the opposite effects [33]. FMR has also been shown to activate SIRT1, reducing oxidative stress to protect cells [34]. The activation of SIRT1 by FMR reduces inflammation and mucus formation, while treatment with an SIRT1 inhibitor reverses the effect of FMR in IL-13-induced inflammation and mucus formation in JME/CF15 cells [35]. These findings are interesting because like SIRT3, SIRT1 is also an important deacetylated protein [36]. They share common acetylated substrate proteins, such as FoxO3a, NF-κB, and PGC-1α. Moreover, they can attenuate liver fibrosis via regulating the AMPK pathway [37,38]. Specially, both SIRT1 and SIRT3 stimulate the nuclear transcription factor FoxO3a [39], modulate NF-κB to decrease inflammation [40], and regulate PGC-1α to reduce lipid accumulation and mitochondrial dysfunction [41]. Identification of the underlying mechanism of the interaction between AST-IV or FMR with SIRT3 would be of great interest.

The present study aimed to evaluate the hepatoprotective potential of the other two major bioactive agents of AST-IV and FMR (chemical structure shown in Figure 1A and B) against oxidative injury in *tert*-butyl hydroperoxide (*t*-BHP)-injured AML12 hepatocytes and to determine whether this effect occurs via activating SIRT3. We demonstrated the novel important role of the SIRT3 modulators AST-IV and FMR in protecting hepatocytes from oxidative damage induced by *t*-BHP.

## 3. Results

### 3.1. AST-IV and FMR Induced SIRT3 Activity and Expression in AML12 Cells

**Figure 2:**
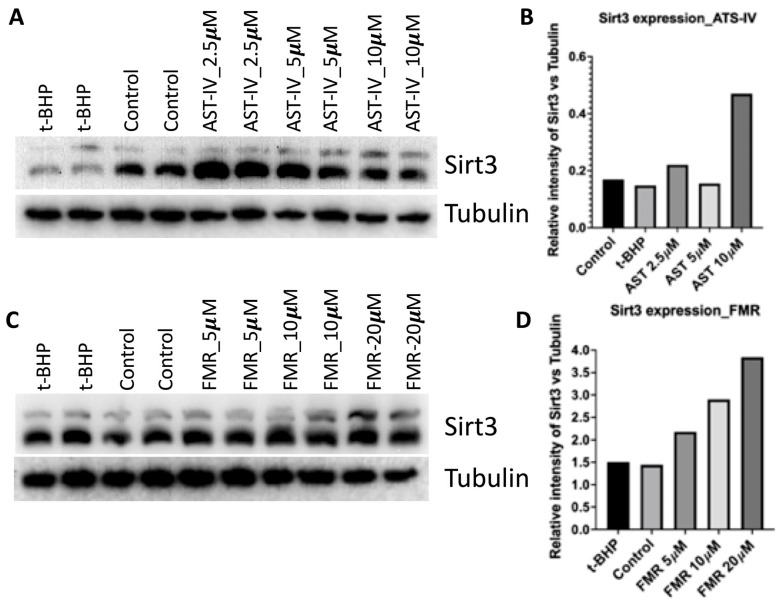
SIRT3 expression (2 isoforms) levels in AML12 cells at different concentrations of AST-IV and FMR. (**A**) Expression of SIRT3 and (**B**) quantification of SIRT3 expression intensity by WB in cells treated with AST-IV at concentrations of 2.5, 5, and 10 µM compared to controls (untreated cells and cells treated with *t*-BHP). (**C**) Expression of SIRT3 and (**D**) quantification of SIRT3 expression intensity by WB in cells treated with FMR at concentrations of 5, 10, and 20 µM compared to controls (untreated cells and cells treated with *t*-BHP). Tubulin was used as a loading control. Data were assessed from at least three independent experiments.

The observation from western blots was that the SIRT3 protein expression level was increased compared to controls (Figure 2). For AST-IV, the expression was highest at 10 µM (Figure 2A,B). For FMR, the expression of SIRT3 was enhanced in a concentration-dependent manner (Figure 2C,D).

### 3.2. Rescue Effect of AST-IV and FMR on t-BHP-Induced Oxidative Injury in AML12 Hepatocytes Assessed by Proliferation and Viability

#### 3.2.1. Rescue Effects of AST-IV and FMR on Cell Proliferation

**Figure 3:**
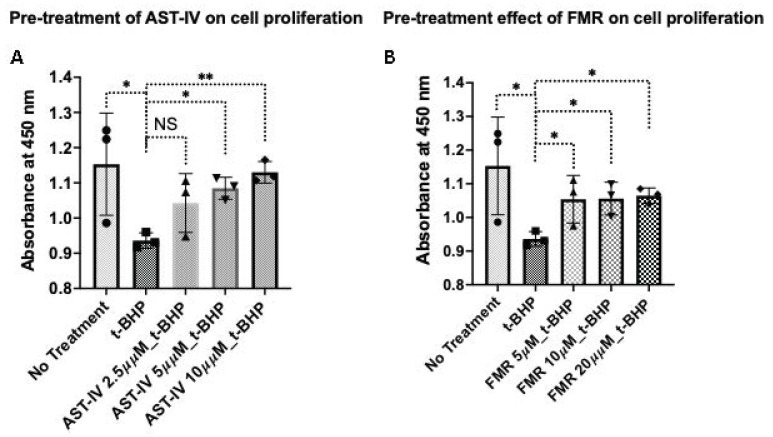
Rescue effect on cell proliferation of AML12 hepatocytes in the condition of pre-treatment with AST-IV or FMR. Rescue effect of AST-IV or FMR on cell proliferation of AML12 hepatocytes in the condition of pre-treatment with AST-IV or FMR for 12 h, then oxidative stress induced by *t*-BHP for another 12 h. (**A**) Effects on cell proliferation of AST-IV at concentrations of 2.5, 5, and 10 µM compared to *t*-BHP-treated cells. (**B**) Effects on cell proliferation of FMR at concentrations of 5, 10, and 20 µM compared to *t*-BHP-treated cells. Data were collected from at least three independent experiments. NS: not significant; * *p* < 0.05, ** *p* < 0.01.

**Figure 4:**
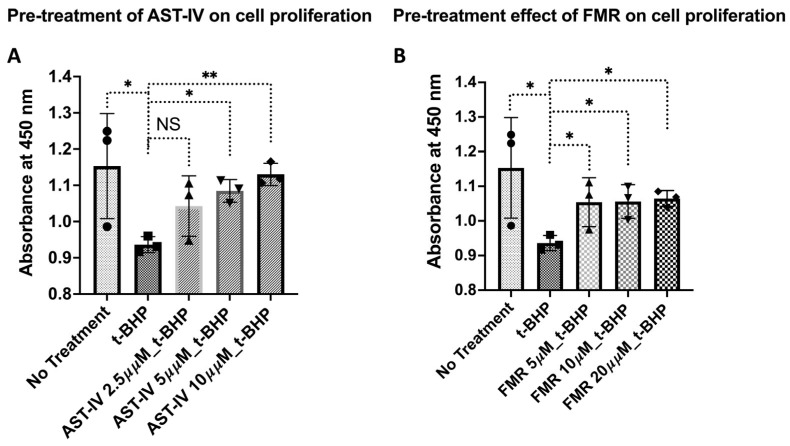
Rescue effect on cell proliferation of AML12 hepatocytes in the condition of post-treatment with AST-IV or FMR. Rescue effect of AST-IV or FMR on cell proliferation of AML12 hepatocytes in the condition of oxidative stress induced by *t*-BHP, then post-treatment with AST-IV or FMR for 12 h. (**A**) Effects on cell proliferation of AST-IV at concentrations of 2.5, 5, and 10 µM compared to *t*-BHP-treated cells. (**B**) Effects on cell proliferation of FMR at concentrations of 5, 10, and 20 µM compared to *t*-BHP-treated cells. Data were collected from at least three independent experiments. NS: not significant; * *p* < 0.05, ** *p* < 0.01.

**Figure 5:**
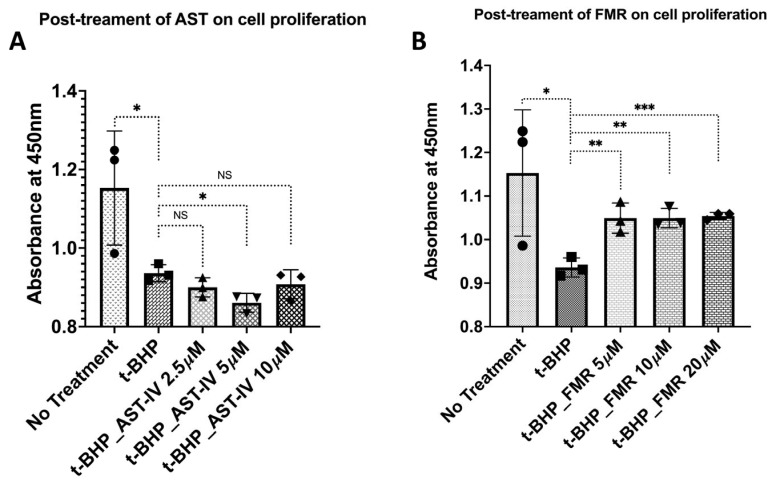
Rescue effect on cell proliferation of AML12 hepatocytes in the condition of combined pre- and post-treatment with AST-IV or FMR. Rescue effect of AST-IV or FMR on cell proliferation of AML12 hepatocytes in the condition of combined pre- and post-treatment with AST-IV or FMR for 12 h, then oxidative stress induced by *t*-BHP for another 12 h. (**A**) Effects on cell proliferation of AST-IV at concentrations of 2.5, 5, and 10 µM compared to *t*-BHP-treated cells. (**B**) Effects on cell proliferation of FMR at concentrations of 5, 10, and 20 µM compared to *t*-BHP-treated cells. Data were collected from at least three independent experiments. NS: not significant, * *p* < 0.05; ** *p* < 0.01, *** *p* < 0.001.

(a) Pre-treatment with AST-IV or FMR: Treatment with AST-IV and FMR showed a significant increase in cell proliferation at higher doses, indicating that AST-IV rescued cell proliferation, which was reduced by *t*-BHP (Figure 3).

(b) Post-treatment with AST-IV or FMR: AST-IV did not show a significant rescue effect; however, FMR showed a pronounced rescue effect on cell proliferation, which was reduced by *t*-BHP, especially at the highest concentration of 20 µM (Figure 4).

(c) Combined pre-treatment and post-treatment (pre- and post-treatment) with AST-IV or FMR: The increase in cell proliferation was pronounced with the combined pre- and post-treatment of AST-IV at concentrations of 2.5, 5, and 10 µM or FMR at 5, 10, and 20 μM compared to cells only treated with *t*-BHP. Higher concentrations of AST-IV or FMR yielded more significant rescue effects on cell proliferation compared to *t*-BHP-treated cells (Figure 5).

#### 3.2.2. Rescue Effects of AST-IV and FMR on ROS Levels

**Figure 6:**
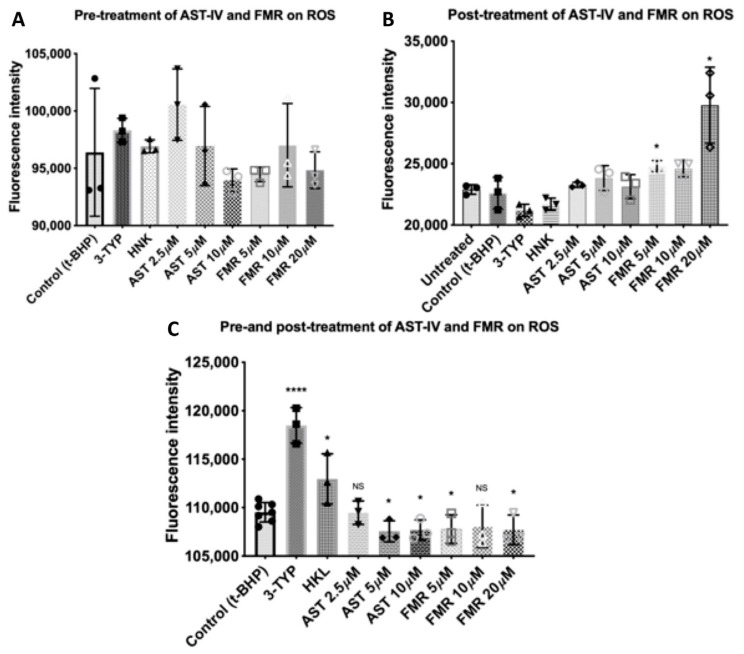
ROS measurement from cells treated with AST-IV or FMR in AML12 hepatocytes in the condition of pre- or post-, or combined pre- and post-treatment with AST-IV or FMR. (**A**) Effects on ROS levels of pre-treatment with AST-IV at concentrations of 2.5, 5, and 10 µM, and FMR at concentrations of 5, 10, and 20 µM, compared to *t*-BHP-treated cells. (**B**) Effects on ROS levels of post-treatment with AST-IV at concentrations of 2.5, 5, and 10 µM, and FMR at concentrations of 5, 10, and 20 µM, compared to *t*-BHP-treated cells. (**C**) Effects on ROS levels of combined pre- and post-treatment with AST-IV at concentrations of 2.5, 5, and 10 µM, and FMR at concentrations of 5, 10, and 20 µM, compared to *t*-BHP-treated cells. Data were collected from at least three independent experiments. NS: not significant, * *p* < 0.05, **** *p* < 0.0001.

AST-IV and FMR showed a significant rescue effect on the ROS level induced by *t*-BHP at concentrations of 5 µM, 10 µM, and 20 µM (Figure 6C) in the combined pre- and post-treatment conditions. In the pre-treatment-only condition or post-treatment-only condition, AST-IV and FMR did not decrease levels of ROS compared to the *t*-BHP-treated cells. In the post-treatment condition, FMR at 5 µM, 10 µM, and 20 µM induced higher ROS levels compared to the control of *t*-BHP-treated cells (Figure 6B). In the pre-treatment condition, the ROS levels were similar in all treated cells compared to the control of *t*-BHP-treated cells (Figure 6A).

### 3.3. Cellular Antioxidant Defense Capacity of AST-IV and Formononetin

#### 3.3.1. SOD Antioxidant Activity

**Figure 7:**
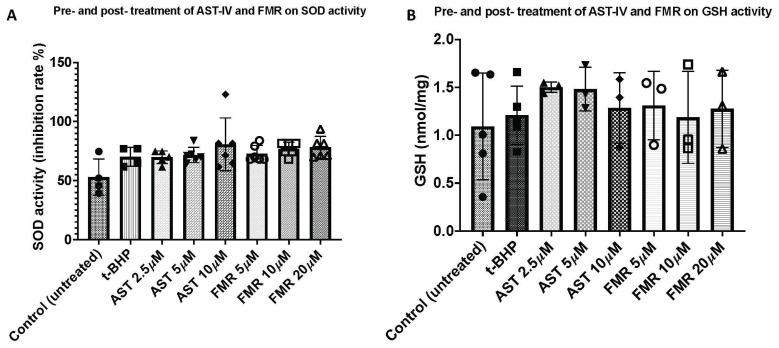
SOD activity and glutathione level measurement from cells treated with AST-IV or FMR in AML12 hepatocytes in the condition of combined pre- and post-treatment with AST-IV or FMR. (**A**) SOD activity in AML12 hepatocytes pre- and post-treatment with various concentrations of AST-IV or FMR; (**B**) glutathione (GSH) level measured in AML12 hepatocytes pre- and post-treatment with various concentrations of AST-IV or FMR. Data were collected from at least three independent experiments.

Prevention of excessive ROS directly relies on antioxidants in cells [42]. Thus, the antioxidative or hepatoprotective capacity of AST-IV and formononetin was examined. We used *t*-BHP at a concentration of 500 µM for inducing oxidative stress in AML12 cells in the treatment condition of combined pre- and post-treatment with AST-IV or FMR, as described in the methods. The antioxidative effects of AST-IV and FMR against *t*-BHP-induced oxidative stress in hepatocytes was determined by measuring superoxide dismutase (SOD) activity. The SOD activity was higher in treated cells, especially AST-IV at 10 µM, compared to controls and *t*-BHP-treated cells (Figure 7A).

#### 3.3.2. Glutathione (GSH) Antioxidant Activity

Glutathione (GSH), a thiol group-containing tripeptide (γ-glutamyl-cysteinyl-glycine), is a key non-enzymatic antioxidant which is capable of reducing ROS-induced damage to important cellular components [12]. Glutathione is present in cells in both reduced (GSH) and oxidized (GSSG) forms. GSH is the predominant species under normal physiological conditions inside cells, while oxidative stress results in increased levels of GSSG [12]. AML12 cells were treated as described above under conditions of combined pre- and post-treatment with AST-IV or FMR and the GSH level was measured using the glutathione assay (BioVision). The GSH level was increased in AST-IV- and FMR-treated cells compared to controls (Figure 7B).

#### 3.3.3. AST-IV and FMR Promote Antioxidant Gene Expression

**Figure 8:**
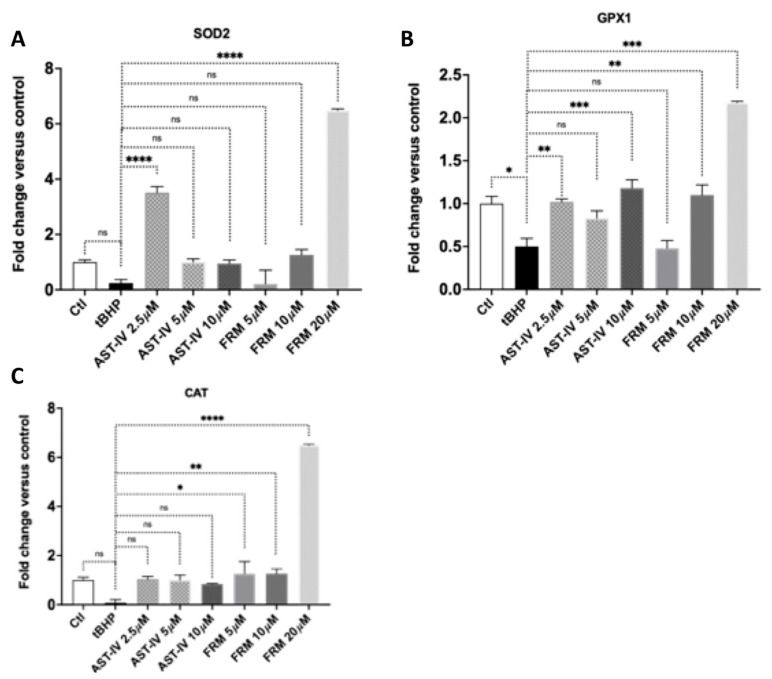
Gene expression of antioxidant genes (**A**) SOD2, (**B**) CAT, and (**C**) GPX1 in AML12 hepatocytes in the condition of combined pre- and post-treatment with AST-IV or FMR. Data were collected from at least three independent experiments and analyzed using Graphpad Prism software version 9.1.1 (223), and statistical analyses using ordinary one-way ANOVA followed by post hoc Dunnett’s multiple comparisons. ns: not significant; * *p* < 0.05, ** *p* < 0.01, *** *p* < 0.001, **** *p* < 0.0001.

AML12 cells were treated in the condition of combined pre- and post-treatment with AST-IV or FMR at various concentrations and harvested for quantitative PCR. Gene expression of the antioxidant gene SOD2 (superoxide dismutase-2) was significantly increased at 2.5 µM of AST-IV and 20 µM of FMR compared to control cells without treatment of AST-IV or FMR. Cat (catalase) and GPX1 (glutathione peroxidase 1) were increased compared to control cells, especially Cat and GPX1 were enhanced significantly at higher concentrations of AST-IV (10 µM) and FMR (10 and 20 µM) (Figure 8).

### 3.4. AST-IV and FMR Prevent Mitochondrial Damage in Oxidation-Injured Hepatocytes via Enhancing Mitochondrial Mass, Membrane Potential, and Biogenesis

Reactive oxygen species damage is associated with a decline in mitochondrial content via mitochondrial biogenesis and function [43]. Pharmacological activation of mitochondrial biogenesis can enhance oxidative metabolism and tissue bioenergetics, and ameliorate mitochondrial dysfunction in acute and chronic diseases [43]. Mitochondrial membrane potential (Δψm) plays a major role in maintaining the physiological function of the mitochondria [44]. Therefore, we evaluated the mitochondrial quantity, mitochondrial potential, and mitochondrial biogenesis of AML12 cells treated with AST-IV or FMR following *t*-BHP-induced oxidative injury. This might be an underlying factor mediating the rescue effect of AST-IV or formononetin on hepatocytes with oxidative injury induced by *t*-BHP.

#### 3.4.1. AST-IV and FMR Enhance Mitochondrial Biogenesis via Activating AMPK and PGC-1α

**Figure 9:**
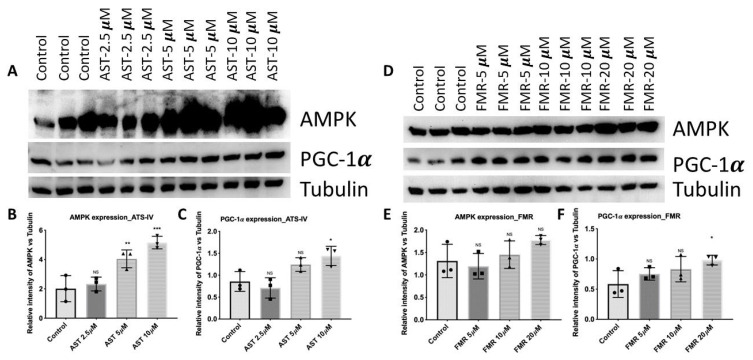
AST-IV and FMR increased mitochondrial biogenesis for their antioxidant effect against oxidative stress induced by t-BHP. (**A**–**C**) Expression and intensity quantification of AMPK and PGC-1α by WB in pre- and post-treatment condition in AML12 cells with AST-IV at concentrations of 2.5, 5, and 10 µM compared to controls (t-BHP-treated cells). (**D**–**F**) Expression and intensity quantification of AMPK and PGC-1α by WB in pre- and post-treatment condition in AML12 cells with FMR at concentrations of 5, 10, and 20 µM compared to controls (t-BHP-treated cells). Tubulin was used as a loading control. Data were assessed from at least three independent experiments. Statistical analyses using ordinary one-way ANOVA followed by post hoc Dunnett’s multiple comparisons. NS: not significant; * *p* < 0.05, ** *p* < 0.01; *** *p* < 0.001.

To identify mitochondrial biogenesis, the mitochondrial master regulator PGC-1α (peroxisome proliferator-activated receptor gamma coactivator 1-alpha) and the phosphorylation level of AMPK (5′ AMP-activated protein kinase) were measured by immunoblotting. AML12 cells were treated under the condition of combined pre- and post-treatment with AST-IV or FMR and collected for western blotting. In cells treated with AST or FMR, PGC-1α and AMPK expression levels were increased, indicating that AST-IV and FMR activated AMPK and PGC-1α for their protective effect against oxidative stress induced by *t*-BHP (Figure 9A,B). Mitochondrial biogenesis was enhanced in AST-IV- and FMR-treated hepatocytes by increasing the mitochondrial biogenesis master regulator PGC-1α and activating AMPK (increased phosphorylated AMPK) (Figure 9A,B). Treatment with AST-IV or FMR resulted in a rescue effect on the decrease in PGC-1α and phosphorylated AMPK caused by *t*-BHP in AML12 hepatocytes. These data solidify the interpretation of a protective effect of AST-IV and FMR on mitochondrial damage in oxidation-injured hepatocytes.

#### 3.4.2. AST-IV and FMR Enhance Mitochondrial Membrane Potential and Mitochondrial Content

**Figure 10:**
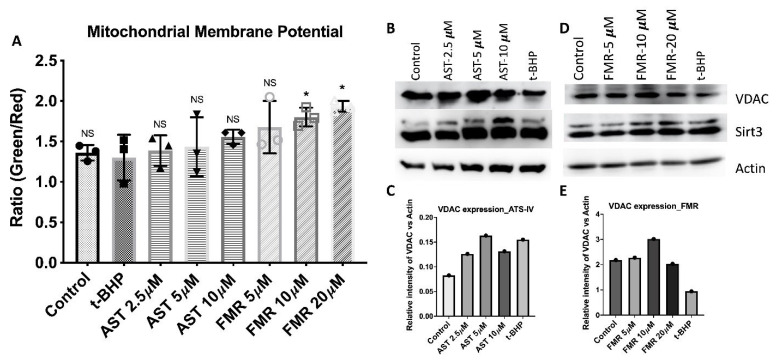
AST-IV and FRM enhance mitochondrial membrane potential and mitochondrial content. AML12 hepatocytes were treated in the condition of combined pre- and post-treatment with AST-IV and FMR, then mitochondrial potential and VDAC were measured. (**A**) Mitochondrial membrane potential of AST-IV- or FMR-treated samples at various concentrations, analyzed using Graphpad Prism Software version 9.1.1 (223), and statistical analyses using ordinary one-way ANOVA followed by post hoc Dunnett’s multiple comparisons. NS: not significant; * *p* < 0.05. (**B**,**C**) Expression of VDAC and SIRT3 (2 isoforms) and VDAC expression quantitation relative to Actin by WB in pre- and post-treatment condition with AST-IV at concentrations of 2.5, 5, and 10 µM compared to controls (untreated cells and *t*-BHP-treated cells). (**D**,**E**) Expression of VDAC and SIRT3 and VDAC expression quantitation relative to Actin by WB in combined pre- and post-treatment condition with FMR at concentrations of 5, 10, and 20 µM compared to controls (untreated cells and *t*-BHP-treated cells) in AML12. Data were assessed from at least three independent experiments.

AML12 hepatocyte cells were treated with AST-IV or FMR in combined pre- and post-treatment conditions and the MMP was measured using the described method. There was an increase in mitochondrial potential Δψm in AST-IV- or FMR-treated hepatocytes compared to untreated hepatocytes or a control, especially at higher concentrations of AST-IV and FMR. This indicates that treatment with AST-IV or FMR resulted in a rescue effect on the decrease in Δψm caused by t-BHP in AML12 hepatocytes (Figure 10A). Mitochondrial content represented by VDAC level also enhanced in treated cells with AST-IV or FMR compared to the control (Figure 10B,C).

## 4. Discussion

In this study, we demonstrated the novel important role of the SIRT3 modulators AST-IV and FMR in protecting hepatocytes from oxidative damage induced by *t*-BHP. The molecular mechanism underlying this hepatoprotective effect of AST-IV and FMR is to activate SIRT3 and the axis SIRT3–AMPK–PGC-1α to enhance mitochondrial biogenesis. Treatment of AST-IV or FMR before and after inducing oxidative stress by *t*-BHP enhanced proliferation, reduced the ROS level, and stimulated the antioxidant defense mechanism as well as enhancing the mitochondrial membrane potential and mitochondrial content.

These results are complementary with the published data on the relationship of AST-IV and FMR with sirtuin members. This makes sense as SIRT3 and SIRT1 work in coordination to regulate metabolic and biological pathways in cells [36]. They share common acetylated protein substrates including FoxO3a, NF-κB, and PGC-1α for reducing inflammation and improving mitochondrial dysfunction as well as being involved in the AMPK pathway for reducing liver fibrosis [39,40]. In addition, they can prevent liver fibrosis through the common AMPK pathway [37,38]. Specifically, both SIRT1 and SIRT3 stimulate the nuclear transcription factor FoxO3a [39], modulate NF-κB to decrease inflammation [40], and regulate PGC-1α to reduce lipid accumulation and mitochondrial dysfunctions [41]. Treatment with AST-IV or FMR significantly up-regulated the VDAC of mitochondrial content and increased the mitochondrial membrane potential to compensate for the reduction in mitochondrial function caused by oxidative stress in liver cells, preventing possible liver inflammation and fibrosis.

It is interesting that several published studies found that AST-IV and FMR activated one member of the sirtuin enzyme group—SIRT1. AST-IV stimulated SIRT1 expression when used on mouse podocytes, which reversed renal fibrosis and improved renal function in the experimental mice compared to mice that were not treated with AST-IV [32]. SIRT1 expression was enhanced, increasing autophagy and improving renal function and fibrosis in mice treated with AST-IV compared to control mice [33]. In addition, FMR was found to activate SIRT1, reducing oxidative stress to protect cells [34]. The stimulation of SIRT1 expression by FMR reduced inflammation and mucus formation while treatment with an SIRT1 inhibitor reversed the effect of FMR in IL-13-induced inflammation and mucus formation in a JME/CF15 cell model [35]. In our study, AST-IV and FMR were identified to stimulate SIRT3, a mitochondrial sirtuin member that regulates most of the metabolic pathways in mitochondria. In addition, the increase in mitochondrial potential and VDAC, depicting mitochondrial content, by treatment with AST-IV or FMR indicated a repair mechanism for damage under conditions of high cellular stress. The increased mitochondrial biogenesis can be coupled with enhanced mitophagy to rapidly replace damaged mitochondria and maintain cellular homeostasis. This rescue effect of AST-IV and FMR implies that they might be used for treatment of liver cell fibrosis or injuries.

Oxidative stress increases levels of reactive oxygen species (ROS) in cells [45]. Oxidative stress can cause damage to cells, potentially destroying tissues [45]. Under normal physiological conditions, there is a balance between ROS and antioxidants in cells to prevent tissue damage and the cells can even use ROS for energy production in the mitochondria [46]. Cells have many mechanisms to maintain that balance. ROS can damage DNA, eventually leading to apoptosis [46]. When cells were treated with *t*-BHP, an agent that causes oxidative stress, experimentally, ROS levels increased and proliferation decreased. However, when cells were treated with AST-IV or FMR before and after induction of oxidative stress, cells were able to reduce the increase in the ROS level and increase cell proliferation. This suggests that AST-IV and FMR have the potential to inhibit the overall oxidative damage in hepatocytes.

Another important cellular defense mechanism against oxidative stress is activation of the antioxidant signaling pathway, which involves activation of most of the antioxidants and detoxifying enzyme genes including catalase (Cat), superoxide dismutase (SOD), and glutathione peroxidase (GPX) [47]. In our study, we reported increased gene expression levels of Cat, Sod2, and GPX1 in hepatocytes treated with AST-IV or FMR at various concentrations. Such a result is consistent with recently published data showing that AST-IV and FMR reduce oxidative stress to protect sciatic nerve tissue [34] and reverse renal fibrosis and improve renal function through regulation of autophagy and podocyte epithelial–mesenchymal transition via activating Sirtuin-1 [32]. Superoxide dismutases (SODs) are ROS scavenging enzymes that catalyze the conversion of superoxide into oxygen and hydrogen peroxide [48], while catalase and glutathione peroxidases allow hydrogen peroxide to form harmless products such as water and oxygen molecules [49]. In this study, we shown the activity of the SOD antioxidant enzyme as well as its transcriptional expression in the combined pre- and post-treatment of AST-IV or FMR in AML12 hepatocytes. Glutathione (GSH) has been a canonical defensive bioagent against oxidative damage caused by drugs, pollutants, or carcinogens [12]. The oxidative stress status can be measured through GSH, which is the reduced form and the predominant species at normal physiological conditions. As oxidative stress increases, the oxidized form (GSSG) becomes the predominant molecule [12]. We determined that glutathione activity was increased in AST-IV- or FMR-treated cells compared to controls. This result, together with increased SOD activity, indicated the cellular antioxidant defense capacity of AST-IV and FMR. The increase in the expression and activity of antioxidant enzymes indicated an increase in the antioxidative capacity of liver cells to control the levels of reactive oxygen species (ROS) and reactive nitrogen species, thus limiting their potential toxicity and the activation of related signaling pathways.

Reactive oxygen species damage is associated with a decline in mitochondrial content via mitochondrial biogenesis and function [43]. Pharmacological activation of mitochondrial biogenesis can enhance oxidative metabolism and tissue bioenergetics, and ameliorate mitochondrial dysfunction in acute and chronic diseases [43]. The mitochondrial membrane potential (Δψm) plays a major role in maintaining the physiological function of the mitochondria [44]. We found that AST-IV and FMR treatments promoted mitochondrial biogenesis via activating AMPK and PGC-1α as well as increasing the mitochondrial membrane potential and mitochondrial content. This might indicate an underlying mechanism mediating the rescue effect of AST-IV or FMR on oxidative injury to hepatocytes induced by *t*-BHP. Furthermore, AST-IV and FMR stimulated SIRT3 expression in *t*-BHP-induced-oxidation-stressed AML12 hepatocytes, especially at a high concentration of FMR. In fact, cells can protect themselves through sirtuins (SirTs), which are deacetylases. Upon DNA damage, SirTs deacetylate the DNA to repress the damaged DNA, preventing the damage from replicating into new cells. SIRT3 is the major deacetylase in the mitochondria and is involved with metabolic homeostasis [50]. When ROS are produced by the mitochondria, SIRT3 neutralizes the ROS or increases the transcription of genes that respond to oxidative stress [51]. This can be used to explain our findings that the activation of SIRT3 by AST-IV or FMR results in rescuing effects on cell proliferation, ROS scavenging, and increased antioxidant activity after inducing oxidative stress in the hepatocytes. From these results, for a next direction, an in vivo study using a mouse model will be conducted to evaluate the protective effects of AST-IV and FMR on induced liver injury.

Taken together, AST-IV and FMR yielded an antioxidant effect, protecting hepatocytes against oxidative stress through induction of SIRT3 protein expression and activation of the antioxidant mechanism as well as mitochondrial biogenesis and function. The findings suggest that these agents, as SIRT3 modulators, could potentially be used in treating oxidation-injury hepatocytes and liver diseases as well as liver toxicity caused by side effects of therapeutic medications.

## 5. Conclusions

In summary, AST-IV and FMR are two potential pharmacologic agents that could protect hepatocytes from oxidative damage in induced oxidative stress in AML12 hepatocytes. The antioxidative protective effect of AST-IV and FMR on liver cells is due to their enhancement of mitochondrial biogenesis and mitochondrial activity via activating PGC-1α and AMPK as well as SIRT3. The findings suggest that these agents, as SIRT3 modulators, can be used in preventing or treating oxidative injury to hepatocytes caused by major pathogenesis of various chronic liver diseases, ethanol cellular injury, and chronic hepatitis.

## 6. Materials and Methods

### 6.1. Electronic Laboratory Notebook Was Not Used

#### Testing Agents

Astragaloside (AST-IV) was purchased from Sigma (Cat # 74777-1MG). It is a cycloartane-type triterpene glycoside (Figure 1A). Formononetin (FMR) was purchased from Selleckchem (Cat # S2299). Formononetin is an O-methylated isoflavone (Figure 1B). *t*-BHP was purchased from Sigma (Cat # 458139-25ML) in liquid. AST-IV and FMR stock solutions were prepared following instructions from the vendor. Accordingly, DMSO was used to assist the dissolution of AST-IV and FMR, and controls with DMSO were included in the experiments.

### 6.2. Cell Culture and Treatment Conditions

AML12 hepatocytes were treated with AST-IV at concentrations of 1, 5, and 10 μM and FMR at concentrations of 5, 10, and 20 μM on *t*-BHP-induced oxidative injury. In the rescue effect experiments, AML12 hepatocytes were treated under 1 of 3 separate conditions unless otherwise stated. Each set of conditions included control groups (untreated and only treatment with *t*-BHP) and AST-IV at concentrations of 2.5, 5, and 10 μM or FMR at concentrations of 5, 10, and 20 μM

Pre-treatment with AST-IV or formononetin: AML12 cells were seeded at 2 × 10^5^ per well in 6-well plates. The cells were treated with various concentrations of AST-IV or FMR and incubated for 12 h. The cells were then incubated with 500 µM *t*-BHP for another 12 h.

Post-treatment with AST-IV or formononetin: AML12 cells were first incubated with *t*-BHP for 12 h, then treated with various concentrations of AST-IV or FMR and incubated for another 12 h.

Both pre-treatment and post-treatment (pre- and post-treatment) with AST-IV or FMR: AML12 cells were treated with various concentrations of AST-IV or FMR and incubated for 12 h. The cells were then incubated with 500 µM *t*-BHP for another 12 h. The cells were further incubated with AST-IV or FMR at the same concentrations as the pretreatment and incubated for another 12 h.

### 6.3. Cell Proliferation and Viability Assay

AML12 hepatocyte cells were seeded to a concentration of 2 × 10^4^ per well in a 96-well plate. The cells were then treated under 3 separate conditions, as described previously, and assessed for cell proliferation and viability. Cell proliferation and cytotoxicity was tested using a Dojindo CCK-8 kit (Cat # CK04-01, Rockville, MD, USA). A volume of 10 µL of CCK-8 solution was added to each well. The plate was then incubated for 3 h at 37 °C. Measurements were made at an absorbance of 450 nm using a microplate reader.

### 6.4. ROS Measurement

ROS measurements were performed using a fluorometric intracellular ROS kit from Sigma (Cat # MAK144, St. Louis, MO, USA). Cells were seeded with a concentration of 2 × 10^4^ per well in a 96-well plate. Cells were then treated with pre- and post-treatment of AST-IV or FMR. When cells were ready to be used, the ROS level was measured following the vendor’s instructions. Fluorescence was measured at EX/EM = 540/570 nm in endpoint mode.

### 6.5. SOD Activity Assay

SOD measurements were performed using a BioVision SOD activity measurement kit (Cat # K335, Milpitas, CA). Cells were seeded and then treated with pre- and post-treatment of AST-IV or FMR. SOD activity in the samples was measured and calculated following the vendor’s instructions.

SOD activity was measured using an SOD activity colorimetric assay. AML12 cells were treated as per the above-described strategies. The cells were then incubated with WST-1 at concentrations and conditions according to the manufacturer’s protocol. WST-1 produces a water-soluble formazan dye upon reduction with the superoxide anion. The rate of the reduction with the superoxide anion is linearly related to the xanthine oxidase (XO) activity and is inhibited by SOD. Therefore, the inhibition activity of SOD was determined by a colorimetric method measuring absorbance at 450 nm using a microplate reader.

### 6.6. Glutathione GSH Measurement

Glutathione GSH measurements were performed using BioVision Reduced Glutathione (GSH) Assay Kit (Cat # K464, Milpitas, CA, USA). Cells were seeded and treated as in pre- and post-treatment with AST-IV and FMR. The GSH level was measured following the instructions from the manufacturer. The media were removed, and cells were washed with PBS. Cells were lysed with lysis buffer. The supernatant was collected, and samples were prepared and the GSH level determined. The assay is based on an enzymatic cycling method in the presence of GSH and a chromophore. The reduction of the chromophore produces a stable product, which can be measured the absorbance at 450 nm using a microplate reader. The absorbance is directly proportional to the amount of GSH in the sample.

### 6.7. Immunoblotting

AML12 hepatocytes treated under various conditions were harvested and lysed with RIPA buffer (supplemented with proteinase and phosphatase inhibitors). Protein concentration standardization was performed using a BCA assay. Cell lysates were then run through an SDS-Page gel followed by immunoblot analysis with specific antibodies (Cell Signaling Technology) against AMPK (5′ AMP-activated protein kinase), PGC-1α (peroxisome proliferator-activated receptor gamma coactivator 1-alpha), SIRT3, VDAC (voltage-dependent anion channel), and Actin or Tubulin as a loading control.

### 6.8. RNA Extraction and Real-Time PCR

Treated AML12 cells were harvested, followed by RNA extraction using RNA STAT60 and subsequent purification using the PureLink RNA Mini Kit (Invitrogen, Waltham, MA, USA). cDNA was synthesized using TaqMan Reverse Transcriptase reagents and random hexamer primers according to the recommendations of the manufacturer. Gene amplification using primers (Table 1) was measured with SYBR Blue using the CFX Connect™ Real-Time PCR Detection System. All reactions were performed in triplicate. After data collection, the mRNA total RNA was calculated with a standard curve generated with serially diluted plasmids containing PCR amplicon sequences and normalized to total RNA with GAPDH as an internal control for assessment.

### 6.9. Mitochondrial Membrane Potential

To measure mitochondrial potential (Δψm), a homogeneous cell-based assay with a water-soluble mitochondrial membrane potential indicator (m-MPI, Codex BioSolutions), as a modified version of JC-1, with similar fluorescent properties and subcellular staining patterns, was used. Fluorescence excitation/emission maxima: 514/529 nm for monomer form; 585/590 nm for J-aggregate form. m-MPI remains in cytoplasm as the monomeric form that shows green fluorescence (emission at 535 nm). The dye undergoes a change in fluorescence emission from green to red when the Δψm increases or vice versa.

### 6.10. Statistics

Statistical analysis was performed using a two-tailed unpaired t-test or one-way analysis of variance analysis (ANOVA) by the Graphpad prism software version 9.1.1 (223). Each experiment was repeated at least 3 times. Results are presented as mean ± S.D. All the statistical details of the experiments can be found in the figure legends, including number of repetitions. *p*-values < 0.05 were considered to indicate statistical significance. Asterisks (*) are used to denote level of statistical significance: * *p* < 0.05; ** *p* < 0.01; *** *p* < 0.001.

## Figures and Tables

**Table 1:** List of primers used in the study.

Gene	NCBI Ref. Seq. (NM)	Primers	Primer Sequences (5′ -> 3′)
SOD2	NM_013671	F	TGG ACA AAC CTG AGC CCT AAG
		R	CCC AAA GTC ACG CTT GAT AGC
CAT	NM_009804	F	GGA GGC GGG AAC CCA ATA G
		R	GTG TGC CAT CTC GTC AGT GAA
GPX1	NM_008160	F	CCA CCG TGT ATG CCT TCT CC
		R	AGA GAG ACG CGA CAT TCT CAA T

## Data Availability

The data used to support the findings of this study are included within the article.

## List of Nonstandard Abbreviations

AML12	Alpha mouse liver 12
AMPK	AMP-activated protein kinase
AST-IV	Astragaloside
Cat	Catalase
FMR	Formononetin
GDH	Glutamate dehydrogenase
GSH	Reduced glutathione
GSSG	Oxidized glutathione (glutathione disulfide)
GPX1	Glutathione peroxidase 1
IDH2	Isocitrate dehydrogenase 2
IL-13	Interleukin 13
Keap1	Kelch-like ECH-associated protein 1
MnSOD	Manganese superoxide dismutase
NAD+	Nicotinamide adenine dinucleotide
NADPH	Nicotinamide adenine dinucleotide phosphate
PGC-1α	Peroxisome proliferator-activated receptor γ coactivator 1α
ROS	Reactive oxygen species
SIRT3	Sirtuin-3
Sirt1	Sirtuin-1
SirT	Sirtuins
SOD	Superoxide dismutase
Sod2	Superoxide dismutase 2
*t*-BHP	*tert*-Butyl hydroperoxide
